# Influence of Non-Alcoholic Fatty Liver Disease on Autonomic Changes Evaluated by the Time Domain, Frequency Domain, and Symbolic Dynamics of Heart Rate Variability

**DOI:** 10.1371/journal.pone.0061803

**Published:** 2013-04-23

**Authors:** Yu-Chen Liu, Chi-Sheng Hung, Yen-Wen Wu, Yi-Chin Lee, Yen-Hung Lin, Chen Lin, Men-Tzung Lo, Chun-Chieh Chan, Hsi-Pin Ma, Yi-Lwun Ho, Chien-Hung Chen

**Affiliations:** 1 Division of Cardiology, Department of Internal Medicine, National Taiwan University Hospital and National Taiwan University College of Medicine, Taipei, Taiwan; 2 Department of Internal Medicine, Far Eastern Poly Clinic, Taipei, Taiwan; 3 Department of Medical Imaging and Cardiology Division of Cardiovascular Medical Center, Far Eastern Memorial Hospital, New Taipei City, Taiwan; 4 National Yang-Ming University School of Medicine, Taipei, Taiwan; 5 Departments of Internal Medicine, National Taiwan University Hospital, Taipei, Taiwan; 6 Division of Biomedical Statistics, Graduate Institute of Epidemiology and Preventive Medicine, College of Public Health, National Taiwan University, Taipei, Taiwan; 7 Center for Dynamical Biomarkers and Translation Medicine, National Central University, Taoyuan, Taiwan; 8 Research Center for Adaptive Data Analysis, National Central University, Taoyuan, Taiwan; 9 Department of Electrical Engineering, National Tsing Hua University, Hsinchu, Taiwan; 10 Division of Gastroenterology, Department of Internal Medicine, National Taiwan University Hospital and National Taiwan University College of Medicine, Taipei, Taiwan; University College London, United Kingdom

## Abstract

**Background:**

Non-alcoholic fatty liver disease (NAFLD) is associated with cardiovascular atherosclerosis independent of classical risk factors. This study investigated the influence of NAFLD on autonomic changes, which is currently unknown.

**Methods:**

Subjects without an overt history of cardiovascular disease were enrolled during health checkups. The subjects diagnosed for NAFLD using ultrasonography underwent 5-min heart rate variability (HRV) measurements that was analyzed using the following indices: (1) the time domain with the standard deviation of N-N (SDNN) intervals and root mean square of successive differences between adjacent N-N intervals (rMSSD); (2) the frequency domain with low frequency (LF) and high frequency (HF) components; and (3) symbolic dynamics analysis. Routine blood biochemistry data and serum leptin levels were analyzed. Homeostasis model assessment of insulin resistance (HOMA-IR) was measured.

**Results:**

Of the 497 subjects (mean age, 46.2 years), 176 (35.4%) had NAFLD. The HRV indices (Ln SDNN, Ln rMSSD, Ln LF, and Ln HF) were significantly decreased in the NAFLD group (3.51 vs 3.62 ms, 3.06 vs 3.22 ms, 5.26 vs 5.49 ms^2^, 4.49 vs 5.21 ms^2^, respectively, all *P*<0.05). Ln SDNN was significantly lower in the NAFLD group after adjustment for age, sex, hypertension, dyslipidemia, metabolic syndrome, body mass index, smoking, estimated glomerular filtration rate, HOMA-IR, and leptin (*P*<0.05). In the symbolic dynamic analysis, 0 V percentage was significantly higher in the NAFLD group (33.8% vs 28.7%, *P* = 0.001) and significantly correlated with linear HRV indices (Ln SDNN, Ln rMSSD, and Ln HF).

**Conclusions:**

NAFLD is associated with decreased Ln SDNN and increased 0 V percentage. The former association was independent of conventional cardiovascular risk factors and serum biomarkers (insulin resistance and leptin). Further risk stratification of autonomic dysfunction with falls or cardiovascular diseases by these HRV parameters is required in patients with NAFLD.

## Introduction

Non-alcoholic fatty liver disease (NAFLD) refers to a spectrum of liver disorders, ranging from simple steatosis to non-alcoholic steatohepatitis, advanced fibrosis, and cirrhosis. [1] The clinical implications of NAFLD stem from its high prevalence, ranging from 10% to 30%, and the potential to progress to liver cirrhosis, hepatic failure, and hepatocellular carcinoma. [2] In Taiwan, the prevalence of NAFLD is 11.5–41% in the adult population, [3] and NAFLD is emerging as an important public health issue worldwide.

The pathogenesis of NAFLD mainly involves insulin resistance and hyperleptinemia, which are also related to atherosclerosis and autonomic changes. [4] Unsurprisingly, cardiovascular disease (CVD) is one of the major causes of death in patients with NAFLD. [5] The association of NAFLD with both carotid and coronary atherosclerosis has been reported to be independent of the classical risk factors for CVD. [6], [7] Conversely, the effect of NAFLD on autonomic changes measured by heart rate variability (HRV) has not been reported. HRV is used widely to assess cardiac autonomic function because of its noninvasiveness and high repeatability. [8] We hypothesized that NAFLD was associated with HRV changes independent of conventional cardiovascular risk factors and serum biomarkers (insulin resistance and leptin). We therefore performed this study to evaluate this issue.

## Materials and Methods

### Study Population

Subjects without an overt history of CVD were enrolled during health checkups at the Far Eastern Poly Clinic from March to November 2011. The exclusion criteria were: past history of CVD (coronary artery disease, myocardial infarction, congestive heart failure, New York Heart Association (NYHA) functional and class, cerebral vascular disease), arrhythmia (atrial fibrillation, premature bigeminy, or trigeminy), pacemaker implant, use of drugs influencing the autonomic system (clonidine, methyldopa, tricyclic antidepressants, scopolamine, antiarrhythmic drugs), hypothyroidism or hyperthyroidism with treatment, thyroid-stimulating hormone (TSH) levels >10 or <0.01 µIU/mL, hemoglobin levels <10 g/dL, or renal insufficiency with estimated glomerular filtration rate (eGFR) <60 mL⋅min^−1⋅^1.73 m^−2^. We defined the group of subjects with NAFLD as the NAFLD group and the group of subjects without NAFLD as the control group. This study was approved by the Ethics Committee of National Taiwan University Hospital. All patients signed the written informed consent.

### Clinical and Laboratory Assessments

Blood pressure was measured after the subjects had rested for 5 min while sitting. Waist circumference was measured at the midpoint between the lower rib and the iliac crest at the end of a normal expiration while the subjects were standing. The body mass index (BMI) was calculated as weight (kg)/height (m^2^). After subjects had fasted for at least 8 h, a blood sample was drawn for analysis of levels of fasting blood glucose and hemoglobin, serum leptin, and plasma total cholesterol, triglyceride, high-density lipoprotein (HDL)-cholesterol, low-density lipoprotein (LDL)-cholesterol, aspartate aminotransferase (AST), alanine aminotransferase (ALT), gamma-glutamyl transpeptidase (GGT), hepatitis B surface antigen (HBsAg), anti-HCV antibody, creatinine, and insulin. Fasting serum insulin levels were measured by an immunochemiluminometric assay (ADVIA Centaur insulin assay, Siemens Healthcare Diagnostic Inc., Tarrytown, NY, USA). Insulin resistance was calculated by the homeostasis model assessment of insulin resistance *(HOMA*-IR index) using the following formula: fasting insulin (µU/mL) level × fasting glucose level (mmol/L)/22.5. Serum leptin level was measured by ELISA using commercial kits according to the manufacturer’s instructions (Human Leptin ELISA kit [K1005-1], B-Bridge international, Inc., Cupertino CA, USA). The inter-assay and intra-assay coefficients of variation of assays were ≤10%. The eGFR (mL·min^−1^·1.73 m^−2^) was calculated using the Modification of Diet in Renal Disease (MDRD) formula: 186× (serum creatinine)^–1.154^×(age)^–0.203^×0.742 (if female).

### Definition of NAFLD

Liver ultrasonography was used to assess the presence of NAFLD. Steatosis was diagnosed based on the presence 1 of the 3 criteria: (1) diffusely increased liver echogenicity with evident contrast between the liver and kidney; (2) diffusely increased liver echogenicity with blurring of the intrahepatic vessels or the diaphragm; (3) bright liver echogenicity with poor penetration of the posterior hepatic segment and intrahepatic vessels or invisibility of the diaphragm. [9] To exclude other causes of liver disease, the subjects with a weekly intake of alcohol >70 g in women and >140 g in men, hepatitis B, hepatitis C, and use of drugs that may induce fatty liver (steroid, amiodarone, estrogen, methotrexate, valproic acid) were excluded from the NAFLD group. [10], [11].

### Definition of Metabolic Syndrome

We used the criteria for the metabolic syndrome proposed by the Third Adult Treatment Panel of National Cholesterol Education Program (NCEP-ATP III). [12] In this study, the defining level of waist circumference was changed to a waist circumference of >90 cm in men and >80 cm in women according to the Asia-Pacific guidelines for managing obesity. [13] Metabolic syndrome was defined clinically, based on the presence of 3 or more of the following: (1) central obesity (waist circumference: men, >90 cm, women, >80 cm, in Asia); (2) a high triglyceride level (>150 mg/dL) or drug treatment for high triglycerides; (3) a low HDL-cholesterol level (men, <40 mg/dL; women, <50 mg/dL) or drug treatment for low HDL-cholesterol; (4) high blood pressure (systolic blood pressure ≥130 mm Hg or diastolic blood pressure ≥85 mm Hg) or antihypertensive drug treatment; (5) a high fasting plasma glucose concentration (>100 mg/dL) or drug treatment for type 2 diabetes.

### Definition of Hypertension and Diabetes Mellitus

Hypertension was defined by systolic blood pressure ≥140 mm Hg or diastolic blood pressure ≥90 mm Hg or on antihypertensive drug treatment. Diabetes mellitus was defined by fasting glucose levels >126 mg/dL, postprandial glucose level >200 mg/dL, or drug treatment for type 2 diabetes.

### Definition of Dyslipidemia

Dyslipidemia was defined based on the presence of ≥3 of the following: (1) a high triglyceride level (>150 mg/dL) or drug treatment for high triglycerides; (2) a high LDL-cholesterol level (>130 mg/dL) or drug treatment for high LDL; (3) a low HDL-cholesterol level (men, <40 mg/dL; women, <50 mg/dL) or on drug treatment for low HDL-cholesterol.

### Measurement of HRV

All subjects underwent 5-min HRV measurements in the supine position in the same room with a fixed room temperature after they had rested for at least 5 min. Recordings were performed using a 24-h ambulatory ECG Holter recording (MyECG E3-80, Mircostar Company, Taipei, Taiwan). An automated algorithm was used for annotating each of the digitalized 1 h ECG data; technicians carefully inspected and corrected the annotated file to enable extraction of the RR intervals.

### Time Domain and Frequency Domain Analysis of HRV

In accordance with the recommendations of the European Society of Cardiology and the North American Society of Pacing Electrophysiology, [8] HRV was analyzed by the following indices: (1) the time domain with the standard deviation of N-N (SDNN) intervals and root mean square of successive differences between adjacent N-N intervals (rMSSD); and (2) the frequency domain with the low frequency (LF: 0.04–0.15 Hz) and high frequency (HF: 0.15–0.4 Hz) components.

### Symbolic Dynamics of HRV

Briefly, consecutive RR interval sequences of at least 300 were selected. Patients with RR interval sequences <300 were excluded. The full range of the sequences was divided into 6 levels (from 0 to 5), and patterns of length L = 3 were constructed. [14] The Shannon entropy of the pattern distributions was calculated to evaluate the complexity of the pattern distribution. [14] All possible patterns were classified into 3 families referred to as: (1) patterns with no variation (0 V; all 3 symbols were equal); (2) patterns with 1 variation (1 V; 2 consequent symbols were equal and the remaining symbol was different); and (3) patterns with 2 variations (2 V; all symbols were different from the previous one). The percentage of the patterns 0 V, 1 V, and 2 V were calculated.

### Statistical Analysis

Based on the report on HRV in patients with metabolic syndrome [15], a sample of 326 participants (163 in each arm) was needed to provide power of at least 90% to detect a significant difference of Ln SDNN between NAFLD and control groups using a 2-sided statistical significance level of P.05, assuming the mean of 3.28, standard deviation of 0.52 in the NAFLD group and mean of 3.46, standard deviation of 0.48 in the control group.

The results are given as mean ±SD or number. The clinical characteristics of the patients were compared by Student’s *t* test for continuous variables and chi-square test for categorical variables. *P*<0.05 was considered statistically significant. Because the distribution of the HRV indices was skewed to the right, the indices were natural logarithm (ln)-transformed, and a normal distribution was confirmed by the Kolmogorov–Smirnov goodness-of-fit test (*P*>0.15). The relationship of NAFLD and HRV indices was investigated by a general linear regression model. *P*<0.05 was considered statistically significant. All statistical analyses were performed using SPSS version 16.0 (Chicago, IL, USA).

## Results

### Characteristics and Clinical Parameters of Study Population

A total of 497 subjects, including 176 men and 321 women aged 18–80 years (mean, 46.2 years), were enrolled for final analysis. There were 176 (35.4%) subjects with NAFLD, and 57 (11.5%) subjects with metabolic syndrome. Compared with the subjects in the control group, subjects in the NAFLD group had higher age, number of men who smoked cigarettes, BMI, waist circumference, systolic and diastolic blood pressure and higher levels of AST, ALT, GGT, total cholesterol, triglyceride, LDL-cholesterol, creatinine, eGFR, fasting glucose, fasting insulin, HOMA-IR, and leptin (all *P*<0.05). Subjects in the control group had higher HDL-cholesterol levels (*P*<0.05). These 2 groups were comparable in TSH, frequency of diabetes, and beta blocker use (all *P*>0.05, Table 1).

**Table 1:** Clinical and biochemical characteristics of participants (N = 497).

Characteristics	NAFLD group (n = 176)	Control group (n = 321)	*P* value
**Age (year**s)	47.6±10.2)	45.4±10.3	0.019
Sex			<0.001
Men	94 (53.4%)	81 (25%)	
Women	82 (46.6%)	243 (75%)	
Body mass index (kg/m^2^)	26.1±3.4	21.8±2.5	<0.001
Waist circumference (cm)	84.6±9.4	72.3±7.8	<0.001
Systolic blood pressure (mm Hg)	124.9±12.8	114.6±12.0	<0.001
Diastolic blood pressure (mm Hg)	76.1±11.5	70.5±9.4	<0.001
AST (IU/L)	22.0±6.7	19.4±5.2	<0.001
ALT(IU/L)	27.4±14.7	16.8±8.3	<0.001
GGT (IU/L)	27.8±20.2	18.0±11.9	<0.001
Fasting glucose (mg/dL)	94.4±11.7	88.1±15.0	<0.001
Total cholesterol (mg/dL)	203.1±33.4	187.3±35.0	<0.001
Triglyceride (mg/dL)	151.3±83.5	88.1±45.0	<0.001
HDL-cholesterol (mg/dL)	49.1±13.4	64.0±15.8	<0.001
LDL-cholesterol (mg/dL)	124.5±36.6	106±32.0	<0.001
Creatinine (mg/dL)	0.7±0.2	0.6±0.2	<0.001
eGFR (mL·min^−1^ **·**1.73 m^−2^)	113.1±25.8	121.5±25.3	<0.001
Fasting insulin (mU/L)	12.14±6.65	7.08±3.71	<0.001
HOMA-IR	2.89±1.80	1.55±0.90	<0.001
Leptin (ng/mL)	16.59±13.38	11.32±8.88	<0.001
Metabolic syndrome	52 (29.55%)	5 (1.54%)	<0.001
Smoking	18 (10.23%)	13 (4.01%)	0.006
Hypertension	40 (22.73%)	23 (7.1%)	<0.001
Diabetes mellitus	6 (3.41%)	7(2.16%)	0.42
Hyperlipidemia	122 (69.32%)	86 (26.54%)	<0.001
Beta blocker user	6 (3.41%)	6 (1.87%)	0.277

Values are expressed as mean ± SD or percentage.

NAFLD, non-alcoholic fatty liver disease; ALT, alanine aminotransferase; AST, aspartate aminotransferase; eGFR, estimated glomerular filtration rate; GGT, gamma-glutamyl transpeptidase.

### Time Domain and Frequency Domain of HRV

The HRV indices of time and frequency domain (Ln SDNN, Ln rMSSD, Ln HF, and Ln HF) were significantly decreased in the NAFLD group (all *P*<0.05, Table 2). These HRV indices were significantly correlated with clinical parameters, insulin resistance, and leptin (only Ln SDNN is shown Table 3). In general linear regression analysis after adjustment for age, sex, hypertension, dyslipidemia, liver function, metabolic syndrome, body mass index, smoking, eGFR, HOMA-IR, and leptin, NAFLD was only negatively associated with Ln SDNN (*P*<0.05) rather than with Ln rMSSD, Ln HF, and Ln LF.

**Table 2:** Comparison of HRV indices between NAFLD subjects and controls.

HRV indices	NAFLD group	Control group	*P* value
	(n = 176)	(n = 321)	
Ln SDNN (ms)	3.50±0.39	3.64±0.38	<0.001
Ln rMSSD (ms)	3.04±0.5	3.23±0.51	<0.001
Ln LF (ms^2^)	5.27±0.99	5.49±1.01	0.021
Ln HF (ms^2^)	4.84±1.07	5.24±1.07	<0.001

Values are expressed as mean ± SD.

NAFLD, non-alcoholic fatty liver disease; HRV, heart rate variability; SDNN, standard deviation of N-N; rMSSD, root mean square of successive differences between adjacent N-N intervals; LF, low frequency; HF, high frequency.

**Table 3:** Pearson’s correlations coefficient between HRV indices and metabolic parameters.

Characteristics	Ln SDNN	0 V	Shannonentropy
Body weight	0.0484	0.1836*	–0.1478*
Body mass index	–0.0623	0.1824*	–0.1469*
Waist circumference	–0.0438	0.2437*	–0.2007*
Aspartate aminotransferase	–0.0749	0.1058*	–0.0441
Alanine aminotransferase	–0.0517	0.1152*	–0.0474
Triglyceride	–0.1769*	0.2180*	–0.1721*
Total cholesterol	–0.1519*	0.1741*	–0.1304*
Uric acid	–0.004	0.2194*	–0.1685*
Blood urea nitrogen	–0.03	0.0143	–0.0175
Creatinine	0.0969*	0.1965*	–0.1657*
eGFR	–0.0164	–0.0042	0.0059
Systolic blood pressure	–0.1318*	0.1995*	–0.1571*
Diastolic blood pressure	–0.1621*	0.1837*	–0.1415*
Fasting blood glucose	–0.1588*	0.1697*	–0.1143*
Insulin	–0.1677*	0.1363*	–0.0737
HOMA-IR	–0.1785*	0.1553*	–0.0885
Leptin	–0.1894*	0.0077	–0.0027

* *P*<0.05.

HRV, heart rate variability; SDNN, standard deviation of N-N; eGFR, glomerular filtration rate; HOMA-IR, homeostasis model assessment of insulin resistance.

### Symbolic Dynamics of HRV

After excluding the patients with RR interval sequences <300, 486 patients underwent symbolic analysis and Shannon entropy calculation (170 in the NAFLD group and 316 in the control group). The 0 V and Shannon entropy were significantly correlated with clinical parameters (Table 3). In the symbolic dynamic analysis, the 0 V percentage was significantly higher in the NAFLD group (33.8% vs 28.7%, *P* = 0.001). 1 V percentage, 2 V percentage, and Shannon entropy were similar in the 2 study groups (Table 4). 0 V was also significantly correlated with linear HRV indices (Ln SDNN, Ln rMSSD, and Ln HF, Table 5). In the multiple regression analysis after adjustment for the age, sex, ALT, fasting glucose levels, hypertension, and total cholesterol levels, 0 V was positively associated with the presence of the metabolic syndrome (*P*<0.05) rather than with NAFLD.

**Table 4:** Comparison of non-linear HRV indices between NAFLD subjects and controls.

HRV indices	NAFLD group	Control group	*P* value
	(n = 170)	(n = 316)	
0 V,%	33.8 (17.1)	28.7(17.3)	0.001
1 V,%	44.8 (9.4)	46.8 (9.1)	0.99
2 V,%	21.4 (11.8)	24.5 (12.3)	0.99
Shannon entropy	3.08 (0.65)	3.2 (0.61)	0.98

Values are expressed as mean ± SD.

NAFLD, non-alcoholic fatty liver disease; HRV, heart rate variability.

**Table 5:** Pearson’s correlation between linear and non-linear indices in NAFLD and control patients.

	Ln SDNN	Ln rMSSD	Ln LF	Ln HF
0 V	–0.1781*	–0.4489*	–0.0485	–0.4462*
1 V	0.0539	0.1132*	0.0247	0.2187*
2 V	0.2128*	0.5537*	0.0504	0.4696*
Shannon entropy	0.1834*	0.3286*	0.0452	0.3234*

* *P*<0.05.

SDNN, standard deviation of N-N; rMSSD, root mean square of successive differences between adjacent N-N intervals; LF, low frequency; HF, high frequency; 0 V, patterns with no variation; 1 V, patterns with 1 variation; 2, patterns with 2 variations.

## Discussion

Our study is the first to demonstrate that NAFLD is associated with autonomic changes evaluated by HRV index. Newton et al. demonstrated cardiac autonomic dysfunction presenting as orthostatic hypotension and a relative nocturnal hypotension in patients with NAFLD. [16] However, there was only 34 subjects in each NAFLD and control groups [16].

### Influences of NAFLD on Time Domain and Frequency Domain Analysis of HRV

As noted earlier, patients with NAFLD have high prevalence rates of the metabolic syndrome. [17] NAFLD might be regarded as a manifestation of the metabolic syndrome and insulin resistance plays a role in both conditions. [1], [11] The components of the metabolic syndrome exert sympathetic activation, [15] and several studies have shown the association between the metabolic syndrome and decreased HRV. [15], [18], [19] Our study also showed that the NAFLD group had a greater frequency of the metabolic syndrome and lower HOMA-IR than the control group. Nevertheless, we demonstrated that NAFLD was associated with autonomic changes (decreased Ln SDNN) independent of traditional CVD risk factors, components of the metabolic syndrome, and HOMA-IR in the present study.

In addition to insulin resistance, the pathogenesis of NAFLD also involves hyperleptinemia, which is also related to atherosclerosis and autonomic changes. [4] Leptin is a hormone that regulates appetite and metabolism. [20] In recent years, leptin has been found to have many biological effects on various systems and may be the signal that integrates vascular, metabolic, and neuroendocrine responses. [21] Plasma leptin levels have been positively associated with cardiovascular complications in humans, [22], [23] and this effect has been observed independent of BMI and traditional cardiac risk factors. [24] Hyperleptinemia is also associated with coronary atherosclerosis, as measured by coronary artery calcium, and this association is independent of insulin resistance. [25] According to Machado et al., leptin level increases progressively with increasing severity of hepatic steatosis. [26] Increasing plasma leptin concentrations are associated with a shift of the sympathovagal balance toward a progressive increase in sympathetic activation. [4] Our study also showed that the NAFLD group had higher plasma leptin levels than the control. However, we also demonstrated that NAFLD was associated with autonomic changes (decreased Ln SDNN) independent of the plasma leptin level in the present study. NAFLD has been shown to have an independent relationship with sub-clinical inflammation. [27] Increased heart rate and reduced HRV are also associated with subclinical inflammation. [28] Therefore, the autonomic changes in NAFLD may be the consequence of subclinical inflammation and increased oxidative stress. Further studies are needed to validate this causal relationship.

### The Association of NAFLD and Symbolic Dynamics Analysis of HRV

The standard linear HRV methods of analysis do not seem adequate to study any short-term instability that may precede major arrhythmias, [14], [29] probably because of the presence of only brief and transient instabilities in the RR interval dynamics [14], [30] that therefore led to controversial results. [14], [29], [31], [32] Guzzetti et al. have proposed a nonlinear method of HRV analysis (3-beat symbolic dynamic analysis) to quantify the prevalence of sympathetic or parasympathetic cardiac modulation in conditions in which the use of a linear HRV approach is limited or disputed. [14] An increase in sympathetic activity results in an increase in 0 V percentage. [14] Our results showed that 0 V percentage was significantly higher in the NAFLD group than in the control group. 0 V percentage was also negatively associated with Ln SDNN in this study. Therefore, both linear and non-linear HRV parameters (Ln SDNN decrease and 0 V percentage increase) indicated activation of sympathetic tones in patients with NAFLD. Conversely, symbolic analysis of 3 beat sequences considers the different time course of sympathetic and parasympathetic cardiac modulations and seems appropriate for elucidating the neural pathophysiological changes in patients with NAFLD. [14] However, 0 V was significantly associated with the presence of the metabolic syndrome rather than with NAFLD in the multiple regression analysis. Therefore, symbolic dynamic analysis can be used for further risk stratification for NAFLD with the metabolic syndrome.

### Clinical Implications

A history of falls is common in NAFLD (43%). [33] Compared with controls, the proportion of people with recurrent falls is significantly higher in NAFLD groups with injuries, emergency medical attention, fracture rates, and hospital admissions. [33] Falls and the aforementioned associations are unrelated to the presence of diabetes or the severity of liver disease. [33] Falls are also considered to be a direct consequence of autonomic nervous system dysfunction. On the other hand, a decrease in SDNN has been reported to be associated with increased overall and cardiovascular mortality in the general population. [34], [35] Symbolic dynamic analysis has been used for the studying short HRV instabilities preceding sudden cardiac death. [36] Our study has demonstrated changes in these parameters in NAFLD. Therefore, further risk stratification of autonomic dysfunction with falls [33] or CVD [37], [38] by these HRV parameters should be studied in patients with NAFLD.

According to the report from Dogru T et al, the circulating levels of asymmetric dimethylarginine (ADMA) are increased in male subjects with biopsy proven NAFLD. [39] The increase of ADMA is independent from traditional cardiovascular risk factors. [39] Li Volti G et al. have proposed a link between ADMA and endothelial dysfunction in db/db mice. After treatment with silibinin in db/db mice, the plasma level of ADMA reduced. The reduction in ADMA level was associated with the improvement of insulin resistance. [40] ADMA has also been reported to evoke sympathetic activation in rat model. [41] Taken together, ADMA may have a role in the pathogenesis of NAFLD, endothelial dysfunction and sympathetic activation. Further researches are warranted to elucidate the connection. Both ADMA and HRV parameters may be regarded as early markers of cardiovascular disease.

### Study Limitations

Our findings have some limitations. First, the design of the study was cross-sectional design; therefore, a causal relationship between NAFLD and changes in HRV parameters could not be demonstrated. Second, NAFLD was defined by ultrasonic examination rather than by tissue biopsy. However, routine tissue biopsy for NAFLD was not feasible in clinical practice.
